# Predictive value of β-catenin in bladder cancer: a systematic review and meta-analysis

**DOI:** 10.1042/BSR20202127

**Published:** 2020-09-04

**Authors:** Jin Ren, Yaodong Yang, Taifang Peng, Dong Xu

**Affiliations:** 1 Department of Urology, The First People’s Hospital of Chongqing Liangjiang New Area, Chongqing 400000, China; 2 Department of Urology, 958 Hospital of PLA Army, Chongqing 400000, China; 3 Department of Urology, Qianjiang Central Hospital of Chongqing, Chongqing 409000, China

**Keywords:** β-catenin, bladder cancer, high grade cancer, Meta-Analysis

## Abstract

Recently, some studies have suggested that the abnormal expression of β-catenin in bladder cancer (BC) is associated with the progression and survival of BC, but there are still some controversies. Hence, we elaborated on the relationship between β-catenin expression and BC through a systematic literature review and meta-analysis. As of March 2020, Embase, PubMed, the Cochrane Library, Science Direct/Elsevier, Medline and CNKI were used for systematic literature retrieval to investigate the correlation between β-catenin expression and BC. Meta-analysis was performed using Review Manager and Stata software. Fourteen studies were included, including 865 BC tissues and 106 controls. Combined ORs were identified with 95% confidence intervals (95% CIs) in a random- or fixed-effects model. We illustrated that there was a significant correlation between β-catenin and BC, that there was abnormally high expression of β-catenin in BC tissues compared with normal bladder tissues (*P*<0.05), and that the combined OR was 14.69 [5.73, 37.65]. Furthermore, the aberrant expression rates of β-catenin in high-grade and invasive bladder neoplasm tissues were greater than those in low-grade and non-muscle-invasive bladder tissues (*P*<0.05), and the combined ORs were 0.31 [0.23, 0.43] and 0.21 [0.15, 0.29]. Finally, we found through meta-analysis that the higher the expression level of β-catenin, the shorter was the progression-free survival (PFS) of patients with BC (*P*<0.05), and the combined OR was 2.74 [1.22, 6.14]. The present study suggests that the abnormal expression of β-catenin is associated with aggressive behavior and poor prognosis of BC, and β-catenin may be a molecular marker of the malignant degree and poor prognosis of BC.

## Introduction

Bladder cancer (BC) is a common malignant tumor of urinary system and one of the ten most common tumors in the whole body, with 77000 new cases in 2016, and an estimated 587426 living with the disease in the U.S.A. [1,2]. The incidence of BC increases with age, with a high incidence among those 50–70 years old. The incidence of BC in males is three- to four-times higher than that in females [3–5]. The etiology and pathogenesis of BC are complex, with both internal genetic factors and external environmental factors. Smoking and occupational exposure to aromatic amine chemicals (such as aniline, 2-aminobiphenyl, 2-naphthalene, 1-naphthalamines etc.) are the two major risk factors for BC [6,7]. At present, some studies suggest that the abnormal function of intercellular adhesion molecules is highly correlated with the invasion of surrounding tissues and distant organ metastasis of BC [8–11]. E-cadherin is an important transmembrane glycoprotein of epithelial cell adhesion. It is also an inhibitor of tumor invasion and metastasis. It plays an important role in maintaining cell morphology, cell movement, and adhesion function [12–14]. E-cadherin needs β-catenin to mediate and participate in its physiological function. β-catenin is an E-cadherin-related protein. The intracellular domain of E-cadherin is connected with β-catenin to form a β-catenin/E-cadherin complex, which is transported to the cell membrane through the cytoplasm and inserted into the cell membrane to maintain the polarity and stability of epithelial cells [15,16].

In the process of tumorigenesis, the abnormality of β-catenin affects the stability of the β-catenin/E-cadherin complex, which leads to a decrease in cell junction density and the loss of cell contact inhibition function. In the development of tumors, it manifests as invasion and metastasis [15–18]. In the study of breast, liver and colon cancer, researchers found that abnormal expression of β-catenin was highly associated with poor prognosis [17–19]. At present, a large number of studies suggest that the aberrant expression of β-catenin is related to the biological characteristics and prognosis of BC, but the results of these studies are inconsistent [20–33]. Some studies have shown that β-catenin is highly expressed in highly invasive BC compared with normal bladder tissues. However, some studies have not found this phenomenon or results. Hence, we elaborated on the relationship between β-catenin expression and BC through a systematic literature review and meta-analysis.

## Materials and methods

### Literature search

Published studies on the correlation of the expression of β-catenin and BC were restricted to a meta-analysis. Two independent researchers retrieved documents from Science Direct/Elsevier, Embase, CNKI, PubMed, the Cochrane Library, and Medline with a search deadline of March 2020 and no language or learning type restrictions. The retrieval terms consisted of MeSH terms and text words. For example, the retrieval terms for β-catenin were ‘beta Catenin’, ‘Catenin, beta’, ‘beta-Catenin’, and ‘β-catenin’, while the search terms for BC were ‘BC’ or ‘Cancer of bladder’ or ‘bladder Neoplasms’ or ‘Urinary Bladder Cancer’ or ‘Neoplasms, bladder’ or ‘Malignant Tumor of Urinary Bladder’ or ‘Urinary Bladder Neoplasms’. All abstracts and relevant documents were retrieved. At the same time, the references in related articles were manually searched, and only full-text documents were included.

### Eligibility criteria

#### Inclusion criteria

The patients were treated with transurethral resection of bladder tumor or radical cystectomy and finally diagnosed with BC by pathological examination. The study groups were staged based on the 2010 American Joint Committee on Cancer guidelines and graded based on the 2004 World Health Organization classification system for transitional cell carcinoma (TCC) [34,35]. The controls were normal bladder tissues. Available data related to the present study were collected from the literature, consisting of the number of aberrant expressions in each group.

#### Exclusion criteria

Case reports, only abstracts, meeting reports, and studies lacking a control population were excluded. Additionally, literature reviews that duplicated previous publications were excluded.

### Paper selection and validity assessment

Literature abstracts and titles that met the inclusion criteria were reviewed by two independent researchers. If the title and abstract of the literature could not be used to judge whether the study should be included, the full text was retrieved for analysis. If there were differences in the literature included, it was assessed by consensus or by a third reviewer. In the meta-analysis, two reviewers conducted quality assessments based on the main criteria for nonrandomized and observational studies of the Newcastle–Ottawa Quality Assessment scale (NOS).

### Data extraction and statistical analysis

Demographic statistics (authors, publishing year, nation, number and age of included study populations) and resulting data of the aberrant expression rate of β-catenin in all studies were collected. Differences were settled by consensus. Two researchers conducted quantitative meta-analyses using RevMan and Stata software. To calculate the combined OR and its 95% confidence interval (95% CI). Heterogeneity was estimated using the *P*-value and the I-square statistic (*I^2^*) in the pooled analyses, which represents the percentage of total variation across studies [36,37]. If the *P*-value was less than 0.1 or the *I^2^*-value was greater than 50%, the summary estimate was analyzed in a random-effects model. Otherwise, a fixed-effects model was applied. In addition, publication bias was detected by visual symmetry of funnel plots, with asymmetry suggesting possible publication bias. This was also assessed by Begg’s and Egger’s tests in the meta-analysis. If the *P*-value was less than 0.05, publication bias existed [36,37].

## Results

### Distinguishing features of inclusion studies

**Figure 1:**
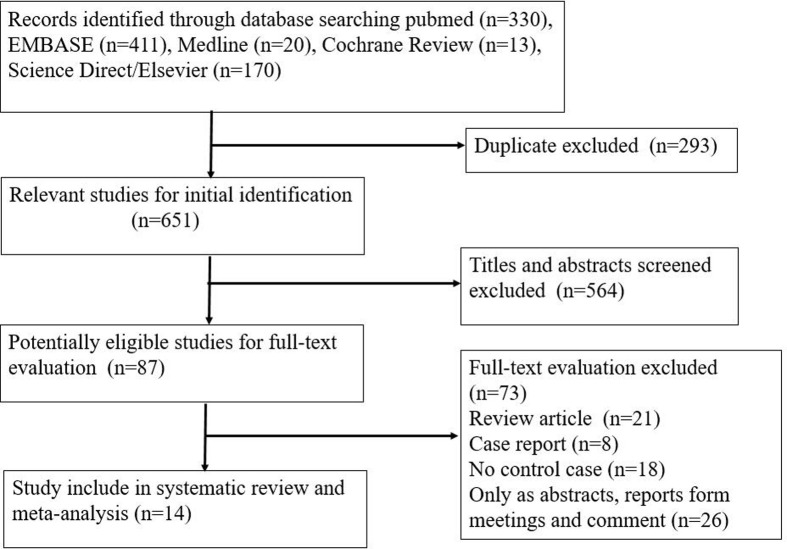
Flow diagram of the selection of eligible studies

The detailed check procedure is exhibited in Figure 1. A total of 651 unduplicated studies were reviewed. According to the criteria of the present study, 14 studies were ultimately screened out. All retrieved studies involved 865 cases of BC tissues and 106 controls. After review by all reviewers, 14 papers were eventually included in the study. The basic data for these 14 studies are exhibited in Table 1. All studies detected β-catenin by streptavidin peroxidase (SP)-conjugated methods. Exclusion/inclusion criteria are as reported in the literature [20–33].

**Table 1:** Characteristics of the included studies

Study	Country	Case	Control	BC	Superficial	Invasive	Low grade	High grade	Assay
Li, 2009	China	52	10/0	52/38	20/9	32/19	42/27	11/11	SP
Dai, 2008	China	73	-	73/36	39/13	34/23	54/19	19/17	SP
Liu, 2005	China	54	11/0	54/40	20/15	30/25	40/28	14/12	SP
Gao, 2014	China	47	25/3	47/23	-	-	21/6	26/17	SP
Kashibuchi, 2007	Japan	-	-	-	20/13	35/21	22/10	33/14	SP
Wang, 2004	China	65	10/0	65/27	28/5	37/22	47/17	18/10	SP
Senol, 2015	Turkey	147	-	-	113/26	34/17	67/11	80/32	SP
Ning, 2014	China	79	-	79/36	42/14	37/22	58/21	21/15	SP
Caria, 2000	China	40	-	40/23	22/4	18/13	16/1	24/16	SP
Elsherif, 2016	Egypt	40	20/14	20/18	-	-	13/1	7/4	SP
Jang, 2010	Korea	80	-	80/19	56/5	24/14	34/2	46/17	SP
Hu, 2011	China	72	20/0	72/38	52/25	20/15	39/16	33/23	SP
Jing, 2014	China	58	-	58/7	45/22	13/9	45/20	18/9	SP
Zhang, 2003	China	58	10	58/31	24/8	34/26	35/12	23/19	SP

### Meta-analysis results

**Figure 2:**
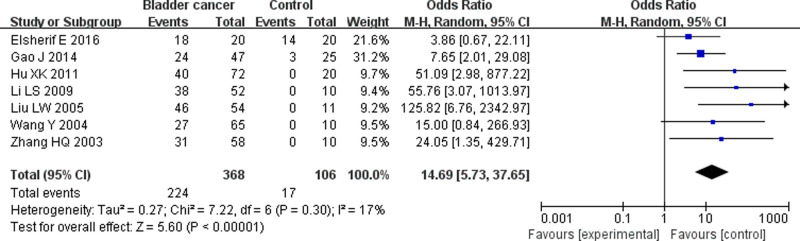
Forest plot showing the meta-analysis outcomes of the aberrant expression rate of β-catenin between BC and normal tissues The aberrant expression rate of β-catenin in BC tissues increased more significantly than that in normal bladder tissues, and the combined OR was 14.69 [5.73, 37.65] (*P*<0.05).

**Figure 3:**
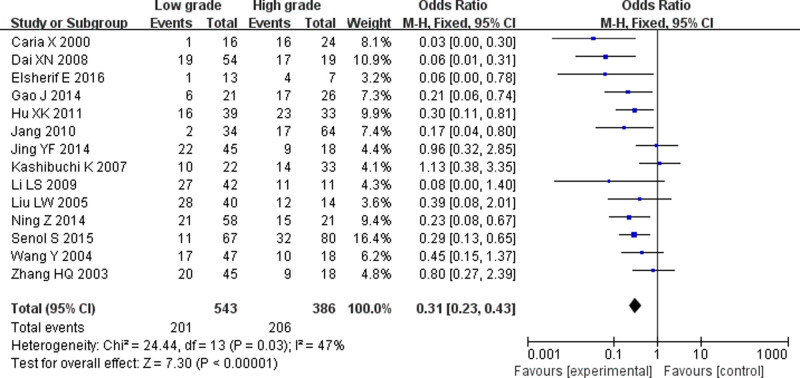
Forest plot showing the meta-analysis outcomes of the aberrant expression rate of β-catenin between high-grade BC and low-grade bladder tissues The aberrant expression rates of β-catenin in high-grade BC tissues were significantly higher than those in low-grade BC tissues, and the combined OR was 0.31 [0.23, 0.43] (*P*<0.05).

**Figure 4:**
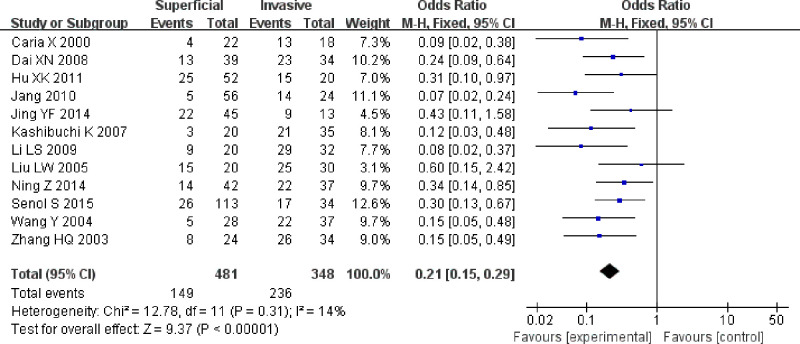
Forest plot showing the meta-analysis outcomes of the aberrant expression rate of β-catenin between invasive BC and non-muscle-invasive bladder tissues The aberrant expression rates of β-catenin in invasive BC tissues were significantly higher than those in superficial bladder tissues, and the combined OR was 0.21 [0.15, 0.29] (*P*<0.05).

**Figure 5:**
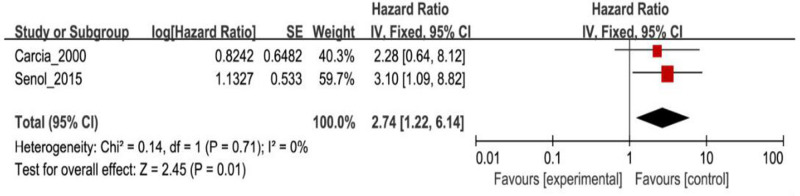
Forest plot showing the meta-analysis of the relationship between the expression of β-catenin and PFS in patients with BC

**Figure 6:**
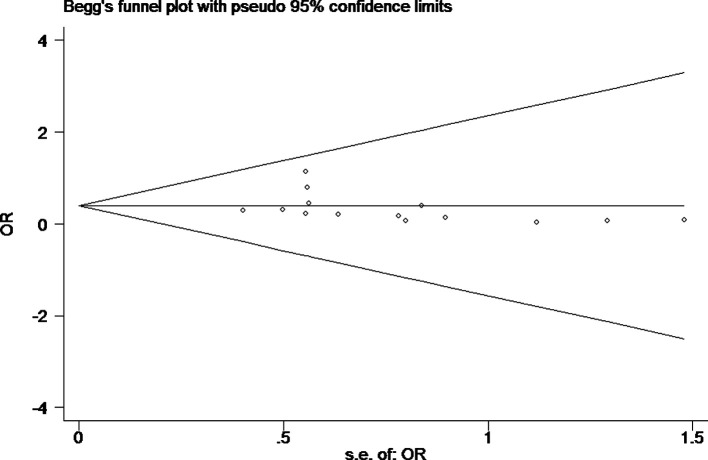
Begg’s publication bias plot of the aberrant expression rate of β-catenin between high-grade BC and low-grade bladder tissues Begg’s funnel plots were roughly symmetrical, suggesting that there was no publication bias in the meta-analysis.

**Figure 7:**
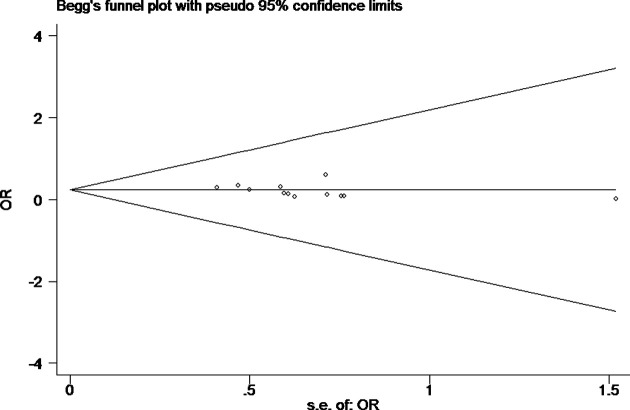
Begg’s publication bias plot of the aberrant expression rate of β-catenin between invasive BC and non-muscle-invasive bladder tissues Begg’s funnel plots were roughly symmetrical, suggesting that there was no publication bias in the meta-analysis.

**Figure 8:**
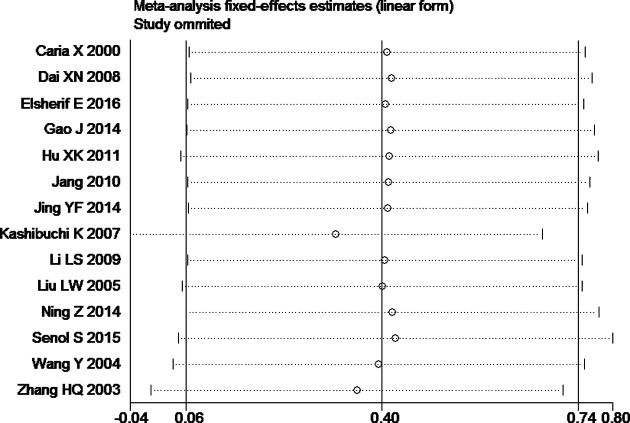
Sensitivity analysis plot of the aberrant expression rate of β-catenin between high-grade BC and low-grade bladder tissues

**Figure 9:**
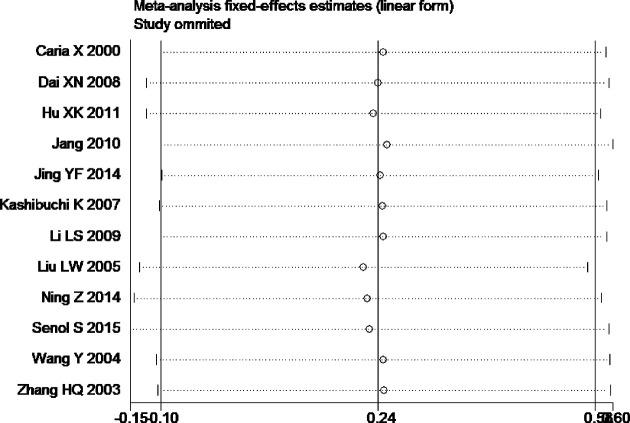
Sensitivity analysis plot of the aberrant expression rate of β-catenin between invasive BC and non-muscle-invasive bladder tissues

Tests for heterogeneity recommended a random-effects model. In our research, a meta-analysis demonstrated that there is a remarkable association between β-catenin expression and BC. Compared with normal bladder tissues, the aberrant expression rate of β-catenin in BC tissues was significantly increased (*P*<0.05), and the combined OR was 14.69 [5.73, 37.65] (Figure 2). Furthermore, the aberrant expression rates of β-catenin in high-grade and invasive BC tissues were notably higher than those in low-grade and non-muscle-invasive BC (NMIBC) tissues (*P*<0.05), and the combined ORs were 0.31 [0.23, 0.43] and 0.21 [0.15, 0.29] (Figures 3 and 4). Finally, we found through meta-analysis that higher the expression level of β-catenin, the shorter the progression-free survival (PFS) of patients with BC (*P*<0.05), and the combined OR was 2.74 [1.22, 6.14] (Figure 5). Begg’s funnel plots suggested that there was no publication bias in the meta-analysis (Figures 6 and 7). Egger’s regression test also indicated little evidence of publication bias (*P*>0.05) (Table 2). In the comprehensive meta-analysis, none of the studies significantly altered the merged results, and the findings indicated that the outcomes were statistically steady and dependable (Figures 8 and 9).

**Table 2:** Egger’s test of publication bias

	Coeff.	Std. Err.	*t*	*P*>|t|	[95% CI]	
High-grade and low-grade	−0.46	0.39	−1.16	0.27	−1.33	0.40
Invasive and superficial	−3.23	0.25	−1.31	0.22	−8.9	0.23

## Discussion

In this work, seven studies reported the anomalous expression rate of β-catenin between BC and normal tissues. Five studies [22,25,27,30,33] reported that the aberrant expression rate of β-catenin in BC tissues was significantly higher than that in normal tissues, and two studies showed that there were no significant differences between BC and normal tissues [21,32]. In this meta-analysis, there was a significant correlation between the aberrant β-catenin expression rate in BC and normal tissues compared with that in normal bladder tissues, and the anomalous expression rate of β-catenin in BC tissues was significantly increased. Fourteen studies examined the aberrant expression rate of β-catenin between low-grade and high-grade BC tissues; eight studies [20,24,26–28,30–32] reported a significant correlation, and six studies showed that there was no significant correlation between the abnormal expression rate of β-catenin and low-grade BC or high-grade BC [21–23,25,29,33]. In this meta-analysis, the abnormal expression rates of β-catenin in high-grade BC were notably higher than those in low-grade BC tissues. Twelve studies examined the aberrant expression rate of β-catenin between non-muscle-invasive and invasive BC tissues; ten studies [20,21,23–29,31] reported a significant correlation, and two studies demonstrated that there was no remarkable correlation between non-muscle-invasive and invasive BC tissues [22,29]. In this meta-analysis, the anomalous expression rates of β-catenin in invasive BC tissues were significantly higher than those in NMIBC tissues.

With the rapid development of urbanization and industrialization and the aggravation of environmental pollution, the incidence and mortality of BC have also increased in recent years. TCC is the most common type of BC. More than 90% of bladder tumors are of this type [38]. BC can be classified as NMIBC (non-muscle-invasive BC) and MIBC (muscle-invasive BC). Approximately 20–30% of newly diagnosed BC is MIBC [39]. The 5-year recurrence rate of NMIBC is as high as 78%, the probability of progression to MIBC is as high as 45%, and the mortality rate of MIBC is as high as 85% in 2 years [40,41]. In view of the high recurrence rate, strong invasion and poor prognosis of BC, it is of great clinical value to actively seek effective biomarkers for the diagnosis, treatment and prognosis of BC. As a multifunctional protein, β-catenin plays an important role in the homeostasis of the tissue environment. Abnormal expression of β-catenin can cause many diseases, such as malignant tumors. In normal physiology, β-catenin can maintain the integrity of epithelial tissue and control the transcription of various extracellular genes. Abnormal expression of β-catenin can induce abnormal signaling pathway activity in normal cells. At this time, β-catenin acts as an oncogene and regulates the transcription of downstream genes to promote the initiation, progression, survival and recurrence of tumors [15–19]. Current studies suggest that Wnt/β-catenin signaling is closely related to the differentiation, proliferation and invasion of BC cells [38–43]. As the core factor of Wnt/β-catenin signaling, β-catenin is involved in many processes, such as the progression, invasion, and repair of BC [37–39].

Accordingly, we collected and analyzed the relevant literature comprehensively and systematically and confirmed that the expression of β-catenin was abnormal in BC. The positive expression level of β-catenin was positively correlated with histological grade and tumor node metastasis (TNM) stage and negatively correlated with patient prognosis. These results suggest that β-catenin mediates the occurrence and evolution of BC. Abnormal positive expression of β-catenin can reflect the clinical stage and pathological grade of BC, and the prognosis of patients is poor. The value of β-catenin is that it can directly provide a simple and accurate reference for clinicians regarding the malignant degree and prognosis of BC.

The present study has the following limitations. First, the sample size is small, and large-scale prospective research is lacking. Second, some literature reference data are incomplete, and research related to this topic is excluded. Furthermore, the 14 papers included in the present study are case–control studies. The quality of evidence is slightly lower in RCT and cohort studies, which limits the quality of the included studies. However, our conclusion has reference value and significance regarding the clinical value for the diagnosis of BC, but in this field, further study is needed.

## Conclusions

This article adds to the proof of a connection between β-catenin and BC. The higher is the expression level of β-catenin, the higher the TNM stage and pathological grade of BC, and the shorter the PFS, suggesting a higher degree of malignancy of BC and a poorer prognosis of patients. β-catenin may be a molecular marker of malignant degree and poor prognosis of BC. However, large-scale in-depth studies can better elucidate the relationship between β-catenin and BC.

## Abbreviations

BC: bladder cancer
*I^2^*: I-square statistic
MIBC: muscle-invasive BC
NMIBC: non-MIBC
PFS: progression-free survival
TCC: transitional cell carcinoma
TNM: tumor node metastasis
95% CI: 95% confidence interval
